# Melatonin in Plants and Plant Culture Systems: Variability, Stability and Efficient Quantification

**DOI:** 10.3389/fpls.2016.01721

**Published:** 2016-11-16

**Authors:** Lauren A. E. Erland, Abhishek Chattopadhyay, Andrew Maxwell P. Jones, Praveen K. Saxena

**Affiliations:** Department of Plant Agriculture, Gosling Institute for Plant Preservation, University of GuelphGuelph, ON, Canada

**Keywords:** degradation, matrix effects, method validation, tissue culture, liquid chromatography–mass spectrometry, serotonin, tryptophan, tryptamine

## Abstract

Despite growing evidence of the importance of melatonin and serotonin in the plant life, there is still much debate over the stability of melatonin, with extraction and analysis methods varying greatly from lab to lab with respect to time, temperature, light levels, extraction solvents, and mechanical disruption. The variability in methodology has created conflicting results that confound the comparison of studies to determine the role of melatonin in plant physiology. We here describe a fully validated method for the quantification of melatonin, serotonin and their biosynthetic precursors: tryptophan, tryptamine and N-acetylserotonin by liquid chromatography single quadrupole mass spectrometry (LC-MS) in diverse plant species and tissues. This method can be performed on a simple and inexpensive platform, and is both rapid and simple to implement. The method has excellent reproducibility and acceptable sensitivity with percent relative standard deviation (%RSD) in all matrices between 1 and 10% and recovery values of 82–113% for all analytes. Instrument detection limits were 24.4 ng/mL, 6.10 ng/mL, 1.52 ng/mL, 6.10 ng/mL, and 95.3 pg/mL, for serotonin, tryptophan, tryptamine, N-acetylserotonin and melatonin respectively. Method detection limits were 1.62 μg/g, 0.407 μg/g, 0.101 μg/g, 0.407 μg/g, and 6.17 ng/g respectively. The optimized method was then utilized to examine the issue of variable stability of melatonin in plant tissue culture systems. Media composition (Murashige and Skoog, Driver and Kuniyuki walnut or Lloyd and McCown's woody plant medium) and light (16 h photoperiod or dark) were found to have no effect on melatonin or serotonin content. A Youden trial suggested temperature as a major factor leading to degradation of melatonin. Both melatonin and serotonin appeared to be stable across the first 10 days in media, melatonin losses reached a mean minimum degradation at 28 days of approximately 90%; serotonin reached a mean minimum value of approximately 60% at 28 days. These results suggest that melatonin and serotonin show considerable stability in plant systems and these indoleamines and related compounds can be used for investigations that span over 3 weeks.

## Introduction

Melatonin (N-acetyl-5-methoxy-tryptamine) is an indoleamine neurohormone, first identified and quantified in plants in 1995 (Dubbels et al., 1995; Hattori et al., 1995). Since then there has been an ever increasing interest in the roles and effects of melatonin in plant systems and it has since been identified as playing important roles in many plant responses including growth, reproduction, development, and stress (Erland et al., 2015; Reiter et al., 2015; Hardeland, 2016). Many of the studies providing insight into these processes rely upon some form of analytical analysis to determine endogenous levels of melatonin in response to a stimulus, treatment or mutation, while treatment often requires prolonged exposure or treatment of plants in *in vitro* culture or greenhouse studies. Validated methods are an essential requirement for accurate quantification of these compounds, and provides both the reader and author confidence in the validity and reproducibility of the data (Betz et al., 2011). Though research methods are available for serotonin and melatonin in plant tissues (Cao et al., 2006; Pape and Lüning, 2006; Garcia-Parrilla et al., 2009; Jiao et al., 2016), most do not also quantify all four of the major phytomelatonin biosynthetic precursors: serotonin, tryptophan, tryptamine and N-acteylserotonin (NAS).

There is controversy in the literature over the stability of melatonin in plants, with both analytical platform, extraction, and analysis methods varying greatly from one report to another with time, temperature, light levels, extraction solvents and mechanical disruption among others all varying widely (Table 1). This has in turn lead to conflicting results between labs, and has contributed to difficulty in confirming and comparing the results across various labs. This is likely, in part, due to the presence of several papers detailing the stability of melatonin from mammalian research (Cavallo and Hassan, 1995; Daya et al., 2001). Another potentially confounding factor in the field of phytomelatonin analysis, is the presence of melatonin isomers in plant products. Recent studies have hypothesized that as many as forty isomers of melatonin may exist in plants, and the presence of these compounds may explain some of this inter-lab variability (Tan et al., 2012; Vigentini et al., 2015). Though oftentimes reports define these compounds as simply “melatonin isomer,” since the initial report of melatonin isomers in wine (Rodriguez-Naranjo et al., 2011), a system of nomenclature has been proposed by Tan et al. (2012), which defines the isomers by the location of the N-acetylaminoethyl and methoxy side chains, and since then several of these theorized isomers have been identified in plant and fermented plant products, though some controversy still exists on this topic (Gomez et al., 2012, 2013; Gardana et al., 2014; Yılmaz et al., 2014; Iriti and Vigentini, 2015).

**Table 1:** **Summary of methods utilized for extraction of melatonin**.

**Sample**	**Amount of melatonin**	**Solvent(s)**	**Freezing or drying**	**Grinding**	**Shaking/ Vortexing**	**Temperature**	**Light**	**Sonication**	**Dry down**	**SPE**	**Total time**	**Analysis type**	**Reference**
*Datura metel* (seed & flower)	<1–250 ng/g	80% methanol	LN	M			Dark	45 min	Under N2		>45 min	LC-MS	Cao et al., 2006; Murch et al., 2009
*Ulva* sp.	7–18 ng/g	100% ethanol		LN	30 min, RT	RT—100°C	Dark		100°C		>85 min	TLC-UV	Tal et al., 2011
*Vitis vinifera* cv Merlot	100–150 μg/g	80% methanol, 1% formic acid	Frozen	M		Ice	Yes	yes			<15 min	LC-MS	Murch et al., 2010
*Vitis vinifera*	4.91–540.12 ng/g	Methanol	N2 gas	M	30 s	15°C	Dim green light	10 min			>15 min	LC-MS	Gomez et al., 2013
Tomatoe (ripe fruit)	4.1–114.5 ng/g	Methanol	Frozen	M				30 min	Vacuum		>30 min	LC-MS	Stürtz et al., 2011
Strawberry (fruit)	2.1–11.26 ng/g	Acetone		M	Yes	25°C		30 min, 25°C	Vacuum	yes	>30 min	LC-MS	Stürtz et al., 2011
Walnut, tomato, sour cherry, green coffee	7.2–341 pg/g	Ethanol	Freeze dried	M	3 min				N2			LC-MS	Kocadağlı et al., 2014
Apple, pear, cherries, bell pepper, plum, tomato, peach, nectarine (fruit)	31.2–521.4 pg/g	Methanol, ethyl acetate	Dry	60 s	15 min		Dim light		Speed-vac, 40°C			LC-MS	Huang and Mazza, 2011
Glycyrrhiza	0.2–34 μg/g	80% ethanol		15 min			Dark				>15 min	LC-UV	Afreen et al., 2006
Barley (*Hordeum vulgar*e)	2–80 ng/g	Chloroform			15 h	4°C	Dark		Speed vac		>15 h	LC-FLD	Arnao and Hernández-Ruiz, 2009
*Echinaceae purpurea* L.	120–300 ng/g	Methanol		M				45 min	Nitrogen gas;		>45 min	LC-MS	Jones et al., 2007
Lupin	5–80 ng/g	Chloroform			15 h	4°C	Dark		Speed vac		>15 h	LC-FLD	Arnao and Hernández-Ruiz, 2013
Lupin	16.2–18.4 ng/g	Ethyl Acetate & butylated hydroxytoluene			15 h	4°C	Dark		Speed vac		>15 h	LC-FLD	Arnao and Hernández-Ruiz, 2007
Rice	0.2–0.8 ng/g	Methanol		LN	Overnight	4°C			Evaporation		>16 h	LC-FLD	Byeon and Back, 2014
Rice	0.5–15 ng/g	Methanol		LN					Evaporation	yes		LC-FLD	Kang et al., 2010
*Chara australis*	2.9–4.5 μg/g	80% Methanol, 1% formic acid	Frozen	M			Red light				<15 min	LC-MS	Lazár et al., 2013
Milk thistle, poppy, anise, coriander, celery, flax, green cardamom, alfalfa, fennel, sunflower, almond, fenugreek, wolf berry, black mustard, white mustard (seeds)	2–189 ng/g	Cold ethanol	Fresh	M		4°C			Vacuum		>10 min	LC-ECD	Manchester et al., 2000
Sunflower	4.6–18.7 μg/g	1 M Tris-HCl, 0.4 M perchloric acid, 0.1% EDTA, 0.05% Na_2_S_2_O_5_, 10 M ascorbic acid	Fresh	LN	1 h	4°C		15 min		yes	>75 min	LC-UV	Mukherjee et al., 2014
St. John's wort	33–549 nmol/g	1 M Tris-HCl; 0.4 M perchloric acid, 0.05% sodium metabissulfate, 0.1% EDTA.		M		RT	Yes				>30 min	LC-ECD-UV	Murch et al., 2000, 2001
Tomatoe	5–200 ng/g	Methanol		M				35 min, 45°C	N2	yes	>55 min	LC-FLD	Sun et al., 2015
Water hyacinth	2.5–20 ng/g	50 mM phosphate buffer, pH 7.4, chloroform		M	5 min		Dim light	20 min	Vacuum		>50 min	LC-MS	Tan et al., 2007
Tomatoe	11–30 ng/g	Methanol	Frozen	LN				35 min, 45°C	N2		>65 min	LC-FLD	Wang et al., 2013
*Nicotiana sylvestris*	13.2–50.4 μg/g	Methanol	Dried	M			Dim light	45 min	N2	yes	>55 min	LC-UV	Zhang et al., 2011
Sweet cherry (*Prunus avium* L. cv Hongdeng)	10–35 ng/g	Methanol	Frozen	LN				35 min, 45°C	N2	yes	>35 min	LC-FLD	Zhao et al., 2013
*Arabidopsis thaliana*	80–120 ng/g	50% methanol	Fresh	M	15 s		Dim green	20 min, 15°C			>50 min	LC-MS	Hernández et al., 2015
Bermudagrass	50–600 pg/g	89% acetone, 10% methanol	Fresh	LN, M					Vacuum	yes	>15 min	ELISA	Shi et al., 2015
*Pyropia yezoensis*	0.15–0.25 ng/g	Chloroform	Frozen	M	Overnight	4°C			Evaporation		>16 h	LC-FLD	Byeon et al., 2014
Tomatoe	2–39.4 ng/g	89% acetone, 10% methanol, 2.5 mM trichloroacetic acid	Frozen	LN	30 min, RT				Vacuum	yes	>45 min	ELISA	Okazaki and Ezura, 2009

ECD—electrochemical detection; ELISA—enzyme linked immunosorbent assay; FLD—fluorescence detection; LC—liquid chromatography; LN—grinding in liquid nitrogen; M—mechanical grinding; MS—mass spectrometry; RT—room temperature; SPE—solid phase extraction; TLC—thin layer chromatography; UV—ultra violet detection.

Additionally, though many reports have now examined the roles serotonin and melatonin play in plants by employing *in vitro* plant tissue culture methods, the actual quantity of melatonin and serotonin which are present in the treatment medium has not been characterized. Induction of cell division, differentiation and morphogenesis in plant cultures are highly sensitive to the relative ratios of plant growth regulators (Skoog and Miller, 1957). Both melatonin and serotonin have the potential to mimic, modulate, and modify auxin and cytokinin ratios in tissues grown *in vitro* (Erland et al., 2015). Variable stability of melatonin and serotonin may lead to a significant difference in their actual content in the medium and within the growing tissues.

This study describes the development of an efficient method for determination of melatonin and its precursors and provides evidence of stability in *in vitro* culture conditions, which may facilitate investigations of regulation of plant development as influenced by interaction of plant hormones.

## Materials and methods

### Sample matrices

Eight species were utilized for validation and three sample types root, shoot (including stems and leaves) and seed for a total of 12 matrices: St. John's wort (*Hypericum perforatum*; SJW) roots and shoots, banana (*Musa* sp.) roots and shoot, African violet (*Saintpaulia* sp.) shoots, potato (*Solanum tuberosum* cv “Shepady”) shoots, sweet wormwood (*Artemisia annua*; *Artemisia*) shoots and roots, tobacco (*Nicotiana tabaccum*) shoots and roots and American elm (*Ulmus americana*) shoots and fennel (*Foeniculum vulgare*) seeds. Fennel seeds were purchased from a local supermarket in Guelph, Ontario, and all other samples were taken from *in vitro* grown cultures maintained at 26°C under a 16 h photoperiod.

### Design of method validation

Accuracy of the method was evaluated by adding known amounts of a given analyte to a given matrix, and the amount ascertained by the method was determined by correcting for endogenous concentrations present in unspiked matrix to determine deviation from the expected value. Precision was evaluated by calculating the relative standard deviation for all measurements for a particular matrix and analyte at each concentration. No fewer than nine determinations were made on 3 different days, with no <2 days separating each set of samples. Accuracy and precision were evaluated across the entire study to ensure method robustness across different days.

Instrument and method limit of detection and limits of quantification were determined according to accepted practices (Bliesner, 2005; AOAC International, 2013), with the limit of detection set to a signal-to-noise ratio of 3:1, and the lower limit of quantitation set to a signal-to-noise of 10:1.

### Sample preparation

For sample preparation prior to analysis samples (~150 mg) were ground in liquid nitrogen then suspended in 0.5 mL of extraction solvent which was comprised of 50% methanol (MS Grade, Fisher Scientific, Canada; MeOH) and 4% acetic acid (glacial, Fisher Scientific, Canada) in Milli-Q water. Extraction solvent was chosen after a literature review (Table 1), and methanol was specifically chosen as it can be directly injected onto a reverse phase chromatography system, as employed in this study, without the need for additional dry down or clean-up steps as required for strong organic solvents such as chloroform or ethyl actetate. Samples were then sonicated (3510R-DTH, Branson, USA) for 15 min on ice and spun down (2 min, 13000 rpm) and, supernatant removed. Supernatant was then filtered through a 0.45 μM centrifuge filter (Millipore; 1 min, 13 0000 rpm) and the flow through was diluted ten times in 10 mM pH 9, adjusted with ammonium hydroxide (Sigma Aldrich, Canada). Prior to analysis samples were either left unspiked or spiked with a high or low concentration of mixed standard containing either 0.5 μg/mL or 5 μg/mL melatonin, serotonin, tryptamine, tryptophan, and NAS, for a total of three sample groups. All standards were analytical grade and purchased from Sigma Aldrich, Canada.

### Detection and quantification

For quantification of samples by liquid chromatography-mass spectrometry, 3 μL of sample was injected onto a Waters Acquity BEH Column (2.1 × 50 mm, i.d. 2.1 mm, 1.7 μm) on a Waters Acquity Classic ultra-performance liquid chromatography (UPLC) system (binary UPLC, Waters, Canada) with single quadrupole mass spectrometer (MS) detection (Waters, QDa performance model, Waters, Canada). Samples were run on a gradient with A—10 mM ammonium acetate pH 9, adjusted with ammonium hydroxide; B—100% MeOH with initial conditions of 95% A 5% B increased to 5% A 95% B over 4.5 min using an Empower curve of 8. Column temperature was 40°C and flow rate was 0.5 mL/min. Compounds were monitored in positive mode in single ion recording (SIR) mode and quantified used standard curves (see Table 2 for MS parameters). In all cases probe temperature was 500°C with a gain of 5.

**Table 2:** **Mass spectrometer parameters for analysis in SIR mode**.

**Analyte**	**m/z**	**Ionization mode**	**Cone voltage**
Serotonin	177	ESI+	10
Tryptophan	205	ESI+	10
Tryptamine	144	ESI+	15
N-acetylserotonin	257	ESI+	5
Melatonin	233	ESI+	15

ESI—electrospray ionization; m/z—mass to charge ratio.

### Youden trial for determination of major factors effecting melatonin stability

Samples were prepared by diluting pure analytical melatonin standard in desired solvent to a concentration of 0.01 mg/mL. Sample were then exposed to levels according to runs designated in Table 3, following a fractional factorial Youden design (Karageorgou and Samanidou, 2014). Factors tested were temperature (4 or 40°C), sonication (0 or 30 min), light (dim green or white), oxidation (bubble through with nitrogen gas 10 s, or not), and solvent (10 or 100% methanol; MeOH). For example, in run one, all samples would be made up in 100% MeOH, prepared under dim green light (green) at 4°C, would be bubbled through with nitrogen gas then left to sit for 30 min without sonication. Organic solvent utilized was pure analytical grade MeOH (Fisher Scientific, Canada) diluted to 10 or 100% with ultra-pure water. For sonication samples not undergoing sonication were held under controlled conditions for an equivalent amount of time without sonication. All runs were conducted using either an ice both or a heated temperature controlled water bath. All runs were repeated in triplicate and conducted one at a time to ensure all samples underwent the same duration of extraction (approximately 40 min). Samples were diluted ten times before being injected (1 μL) for analysis on a Waters Classic Acquity ultra performance liquid chromatography (UPLC) system with electrochemical detection (Coulochem III, ESA, Dionex, ThermoFisher Scientific; ECD) equipped with an ultra-analytical coulometric flow cell (ThermoFisher Scientific, USA). Separation was performed on a Waters BEH Phenyl column (2.1 × 50 mm, 1.7 μm) using an isocratic flow of 75% 100 mM sodium acetate (Sigma Aldrich, Canada) buffer with 100 mM citric acid (Sigma Aldrich, Canada), and 25% MeOH at a rate of 0.4 mL/min with a column temperature of 35°C. Detection was performed with screening voltage of 100 mV, and detection at 850 mV, 1 μA collecting 30 points per second. Melatonin eluted at 3.5 min, limit of detection was determined to be 10 ng/mL and limit of quantification was 30 ng/mL. To calculate effect of the various factors the average percent melatonin concentration remaining in high treatments was subtracted from the average percent melatonin content remaining in the low treatment level. Melatonin standard was purchased from Sigma Aldrich, Canada.

**Table 3:** **Design for Youden trial, in all cases *n* = 3, uppercase letter indicates high level, lowercase letters indicate low level**.

	**Temperature (A/a)**	**Sonication (B/b)**	**Light (C/c)**	**Oxidation (D/d)**	**Solvent (E/e)**
Run 1	4°C (a)	0 min (b)	Green (c)	Nitrogen (d)	100% MeOH (e)
Run 2	40°C (A)	0 min	Green	– (D)	10% MeOH (E)
Run 3	4°C	30 min (B)	Green	–	10% MeOH
Run 4	40°C	30 min	Green	Nitrogen	100% MeOH
Run 5	4°C	0 min	White (C)	Nitrogen	100% MeOH
Run 6	40°C	0 min	White	–	10% MeOH
Run 7	4°C	30 min	White	–	10% MeOH
Run 8	40°C	30 min	White	Nitrogen	100% MeOH

### Media stability

Fifty millimolars Melatonin and serotonin stock solutions were prepared in 96% ethanol (Philips Canada, Scarborough, Ontario), just prior to media sterilization and stored at −20°C until ready for use. For media preparation three media salts were utilized: Murashige and Skoog, Driver and Kunyuki (DKW) and Llyod and McCown's woody plant medium (WPM) with Gamborg B5 vitamins as per the manufacturers recommended concentrations and media were further supplemented with 3% sucrose (Murashige and Skoog, 1962; Gamborg et al., 1968; McCown and Lloyd, 1981; Driver and Kuniyuki, 1984). Media pH was adjusted to 5.7 using 0.1 N sodium hydroxide (Fisher Scientific, Canada) and sterilized by autoclaving for 20 min at 121°C and 21 PSI. Post-autoclaving, media was cooled by incubating in a water bath at 55°C. Melatonin and serotonin were then added to media in an aseptic fashion for a final concentration of 25 μM each. Media were then dispensed into Magenta GA-7 boxes (Caisson Labs, Utah, USA) and further divided into light (~40 μmol m^2^s^−1^) and dark (0 μmol m^2^s^−1^) treatments, each replicated thrice. All boxes were sealed with 3M Micropore tape and were stored in growth rooms maintained at 24 ± 2°C under a 16 h photoperiod provided by cool white fluorescent lamps (Philips Canada, Scarborough, ON). 500 μL samples were collected aseptically at 0 min (immediately after addition of melatonin or serotonin stock to medium), 5 min, 30 min, 1 h, 3 h, 6 h, 12 h, 24 h, 3 days, 10 days, 14 days, 21 days, and 28 days, and flash frozen in liquid nitrogen and stored at −80°C.

To remove media salts and sugar from samples, samples were loaded onto a Waters Oasis HLB solid phase extraction (SPE) cartridge (1 cc, 30 mg, Waters, Canada), samples were then washed with 1 mL of Milli-Q water and eluted in 0.5 mL of 100% MS grade MeOH. Samples were then diluted ten times in Milli-Q water and 5 μL was injected and analyzed following the validated UPLC-MS protocol as described above.

### Data analysis

All data were plotted and analyzed in Microsoft Excel 360 (Microsoft, USA) for experiments performed on the UPLC-ECD system, while all data from UPLC-MS experiments were plotted and analyzed in GraphPad Prism 6. For media analysis treatment groups were compared using a paired two-way ANOVA, with α = 0.05. The Youden trial was designed and analyzed as per the literature, with only five factors included (Karageorgou and Samanidou, 2014). All samples were repeated in triplicate, and all experiments were replicated twice, and data was combined.

## Results

**Figure 1:**
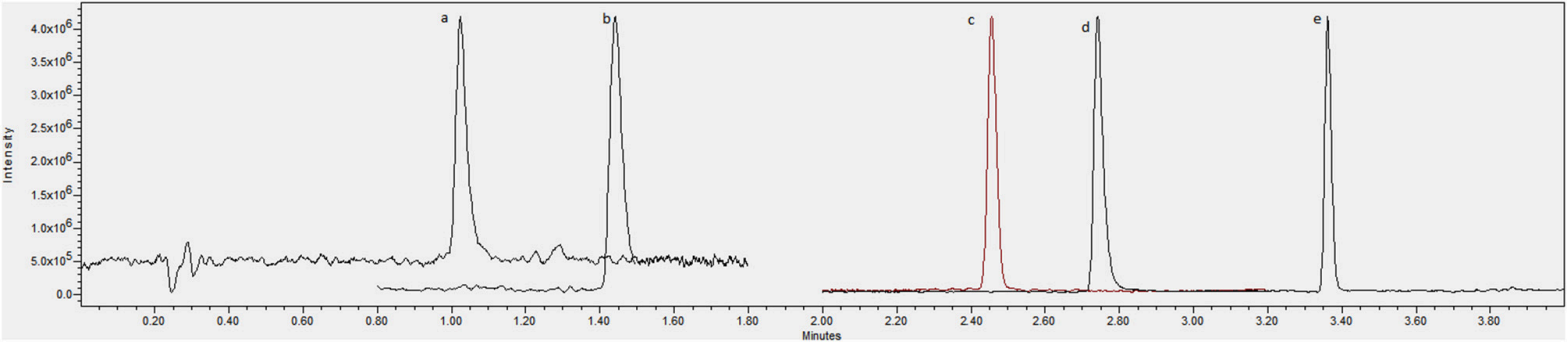
**Chromatogram showing an overlay of channels for (A)** serotonin, **(B)** tryptophan, **(C)** tryptamine, **(D)** N-acetylserotonin and **(E)** melatonin standards at 1 μg/mL.

**Figure 2:**
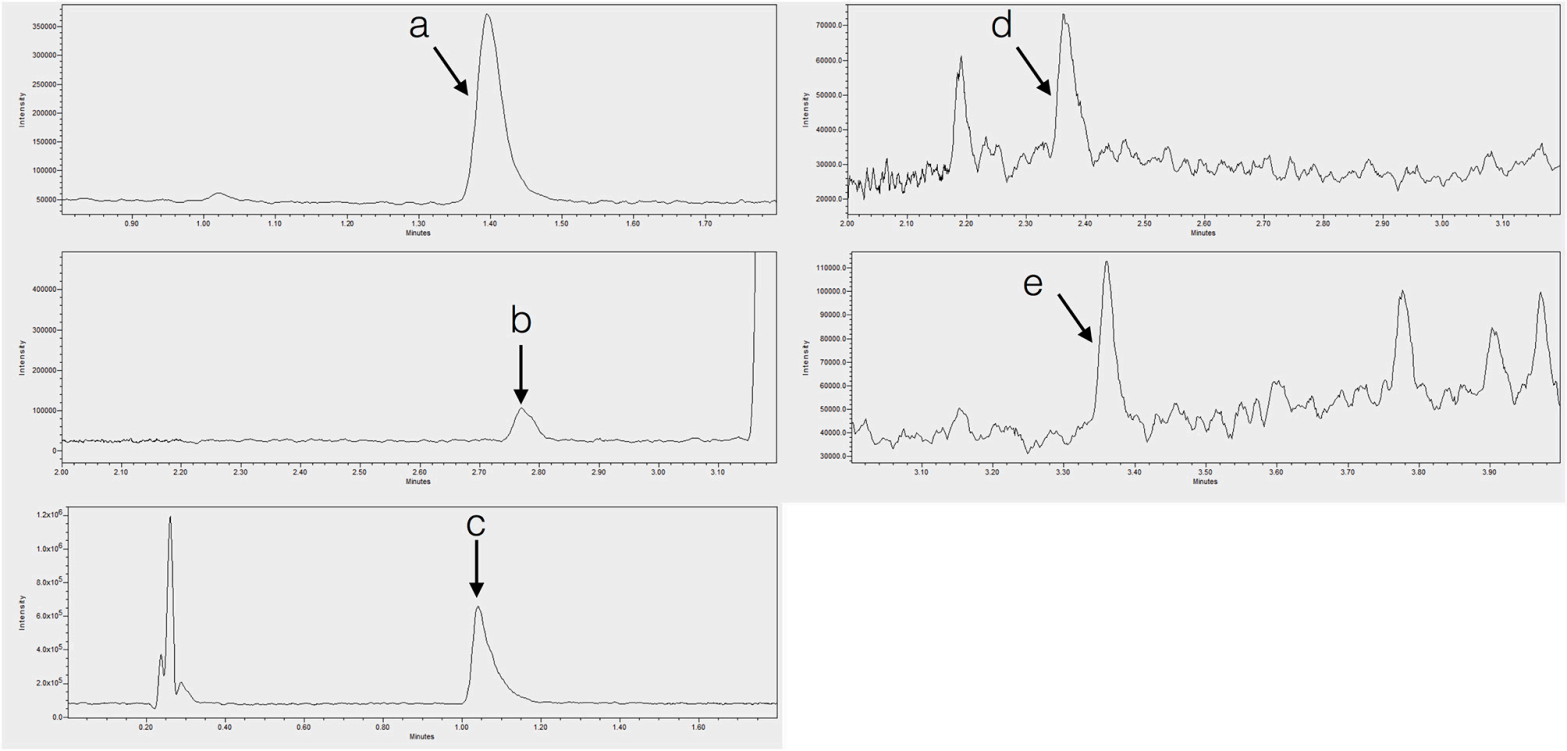
**Chromatograms showing endogenous (A)** tryptophan in tobacco shoot, **(B)** tryptamine in tobacco shoot, **(C)** serotonin in banana root, **(D)** N-acetylserotonin in potato shoot, and **(E)** melatonin in SJW shoot.

The method presented in this paper showed good specificity for all compounds due to the use of a single quadrupole system in SIR mode (Figures 1, 2), with all peaks being completely resolved from surrounding peaks and showing good signal to noise (>3:1) in the linear range. Endogenous concentrations in all matrices are shown in Table 4.

**Table 4:** **Concentrations of tryptophan (Trp), tryptamine (Trm), serotonin (Ser), N-acetylserotonin (NAS) and melatonin (Mel) in tissues studied in this validation**.

**Species**	**Tissue**	**Mean concentration in tissue (standard error)**				
		**Trp (μg/gFW)**	**Trm (μg/g FW)**	**Ser (μg/g FW)**	**NAS (ng/g FW)**	**Mel (ng/g FW)**
SJW	Shoot	164.8 (25.4)	1.51 (0.31)	nd	nd	32.5 (1.70)
	Root	15.2 (2.8)	0.599 (0.057)	nd	nd	9.72 (1.12)
African violet	Shoot	58.9 (18.2)	0.508 (0.085)	nd	nd	nd
Banana	Shoot	91.2 (12.3)	0.627 (0.063)	7.17 (1.68)	nd	16.81 (2.2)
	Root	20.2 (5.0)	0.486 (0.008)	34.17 (6.36)	nd	nd
Elm	Shoot	16.7 (1.7)	nd	nd	nd	nd
Tobacco	Shoot	16.3 (2.2)	0.721 (0.069)	nd	nd	nd
	Root	4.5 (1.9)	nd	nd	77.1 (9.9)	nd
Potato	Shoot	39.1 (5.3)	0.719 (0.17)	nd	330 (86.4)	40.05 (1.85)
Artemisia	Shoot	29.9 (5.9)	nd	nd	nd	nd
	Root	15.1 (1.1)	nd	nd	nd	nd
Fennel	Seed	24.43 (2.73)	0.0733 (0.0126)	nd	nd	33.30 (13.0)

Instrument limits of detection were 24.4 ng/mL, 6.1 ng/mL, 1.52 ng/mL, 6.1 ng/mL, and 92.5 pg/mL for serotonin, tryptophan, tryptamine, NAS and melatonin, respectively. Method detection limits were found to be 1.62 μg/g, 0.407 μg/g, 0.101 μg/g, 0.407 μg/g, and 6.17 ng/g respectively. The linear range (lower limit of quantification; LLOQ–upper limit of quantification; ULOQ) for each analyte was 97.7 ng/mL–25 μg/mL, 24.4 ng/mL–25 μg/mL, 6.1 ng/mL–6.25 μg/mL, 24.4 ng/mL–25 μg/mL and 38.1 pg/mL–6.25 μg/mL for serotonin, tryptophan, tryptamine, NAS, and melatonin respectively, showing a linear range of more than 4 orders of magnitude (Table 5).

**Table 5:** **Summary of retention time, and instrument and method limits of detection and quantification for all analytes investigated**.

**Analyte**	**Retention time (min)**	**Instrument LOD (ng/mL)**	**Method LOD (μg/g)**	**Instrument LLOQ (ng/mL)**	**Method LLOQ (μg/g)**	**Instrument ULOQ (μg/mL)**	**Method ULOQ (mg/g)**
Serotonin	1.057	24.4	1.62	97.7	6.51	25	1.67
Tryptophan	1.045	6.10	0.407	24.4	1.62	25	1.67
Tryptamine	2.752	1.52	0.101	6.1	0.407	6.25	0.42
N-acetylserotonin	2.480	6.10	0.407	24.4	1.62	25	1.67
Melatonin	3.369	0.093	0.00617	0.38	0.0254	6.25	0.42

LOD—limit of detection, LLOQ—lower limit of quantification, RT—retention time, ULOQ—upper limit of quantification.

Excellent reproducibility, presented as percent relative standard deviations (% RSD), was demonstrated for all five analytes in all of the eight matrices ranging from 4–8 and 1–9% in low and high spikes respectively for serotonin; 2–4 and 4–5% for tryptophan; 2–7 and 1–5% for tryptamine; 1–4% for both low and high spikes in N-acetylserotonin and 7–8 and 6–10% for melatonin (Table 6).

**Table 6:** **Recovery data for serotonin, tryptophan, tryptamine, N-acetylserotonin and melatonin**.

**Species**	**Tissue**	**Low Recovery^a^ Average**	**Low Recovery^a^ %RSD**	**High Recovery^b^ Average**	**High Recovery^b^ %RSD**
**SEROTONIN**					
SJW	Shoot	103%	5%	100%	1%
SJW	Root	101%	6%	97%	2%
African Violet	Shoot	101%	5%	100%	3%
Banana	Shoot	102%	7%	111%	8%
Banana	Root	85%	8%	113%	9%
Elm	Shoot	100%	7%	102%	5%
Tobacco	Shoot	100%	7%	98%	1%
Tobacco	Root	100%	5%	101%	1%
Potato	Shoot	110%	5%	116%	2%
Artemisia	Shoot	104%	5%	107%	1%
Artemisia	Root	107%	4%	106%	1%
Fennel	Seed	101%	1%	101%	1%
**TRYPTOPHAN**					
SJW	Shoot	93%	3%	98%	5%
SJW	Root	94%	3%	94%	5%
African Violet	Shoot	93%	4%	96%	5%
Banana	Shoot	90%	4%	97%	5%
Banana	Root	95%	4%	96%	5%
Elm	Shoot	94%	2%	96%	5%
Tobacco	Shoot	101%	5%	97%	5%
Tobacco	Root	96%	3%	95%	5%
Potato	Shoot	97%	2%	100%	4%
Artemisia	Shoot	96%	2%	98%	4%
Artemisia	Root	99%	2%	101%	4%
Fennel	Seed	99%	2%	102%	2%
**TRYPTAMINE**					
SJW	Shoot	99%	2%	101%	2%
SJW	Root	90%	3%	98%	1%
African Violet	Shoot	92%	2%	96%	1%
Banana	Shoot	94%	2%	94%	2%
Banana	Root	106%	7%	100%	2%
Elm	Shoot	92%	3%	103%	2%
Tobacco	Shoot	98%	3%	104%	2%
Tobacco	Root	98%	5%	104%	2%
Potato	Shoot	106%	2%	105%	2%
Artemisia	Shoot	86%	7%	82%	5%
Artemisia	Root	110%	7%	104%	2%
Fennel	Seed	110%	3%	99%	2%
**N-ACETYLSEROTONIN**					
SJW	Shoot	97%	4%	96%	3%
SJW	Root	97%	1%	99%	2%
African Violet	Shoot	100%	1%	95%	1%
Banana	Shoot	104%	2%	98%	1%
Banana	Root	104%	2%	99%	2%
Elm	Shoot	99%	1%	100%	2%
Tobacco	Shoot	99%	2%	97%	3%
Tobacco	Root	102%	2%	99%	2%
Potato	Shoot	98%	1%	97%	2%
Artemisia	Shoot	93%	2%	94%	1%
Artemisia	Root	95%	3%	101%	4%
Fennel	Seed	110%	3%	90%	2%
**MELATONIN**					
SJW	Shoot	96%	7%	92%	6%
SJW	Root	100%	8%	95%	6%
African Violet	Shoot	98%	7%	96%	6%
Banana	Shoot	98%	7%	97%	6%
Banana	Root	106%	8%	97%	6%
Elm	Shoot	99%	7%	98%	6%
Tobacco	Shoot	101%	7%	98%	7%
Tobacco	Root	104%	7%	89%	10%
Potato	Shoot	106%	7%	100%	7%
Artemisia	Shoot	102%	7%	98%	7%
Artemisia	Root	113%	8%	105%	6%
Fennel	Seed	94%	1%	98%	5%

a *low spike concentration was 0.5 μg/mL*.

b *high spike concentration was 5 μg/mL*.

Recovery was also acceptable for all matrices and analytes with low concentration spike recoveries ranging from 85% in banana root to 110% in potato shoot for serotonin; 93% in SJW shoot to 101% in tobacco shoot for tryptophan; 90% in SJW root to 110% in *Artemisia* root for tryptamine; 93% in *Artemisia* shoot to 110% in fennel seed for NAS and 94% in fennel seed to 113% in *Artemisia* root for melatonin. At high concentration recoveries were similar with values of 97–113% for serotonin (SJW root, and banana root); 94–101% for tryptophan (SJW and *Artemisia* root); 82–104% for tryptamine (SJW and *Artemisia* root); 90–101% for NAS (fennel seed and root); and 92–105% for melatonin (SJW shoot and *Artemisia* root) (Table 6).

**Figure 3:**
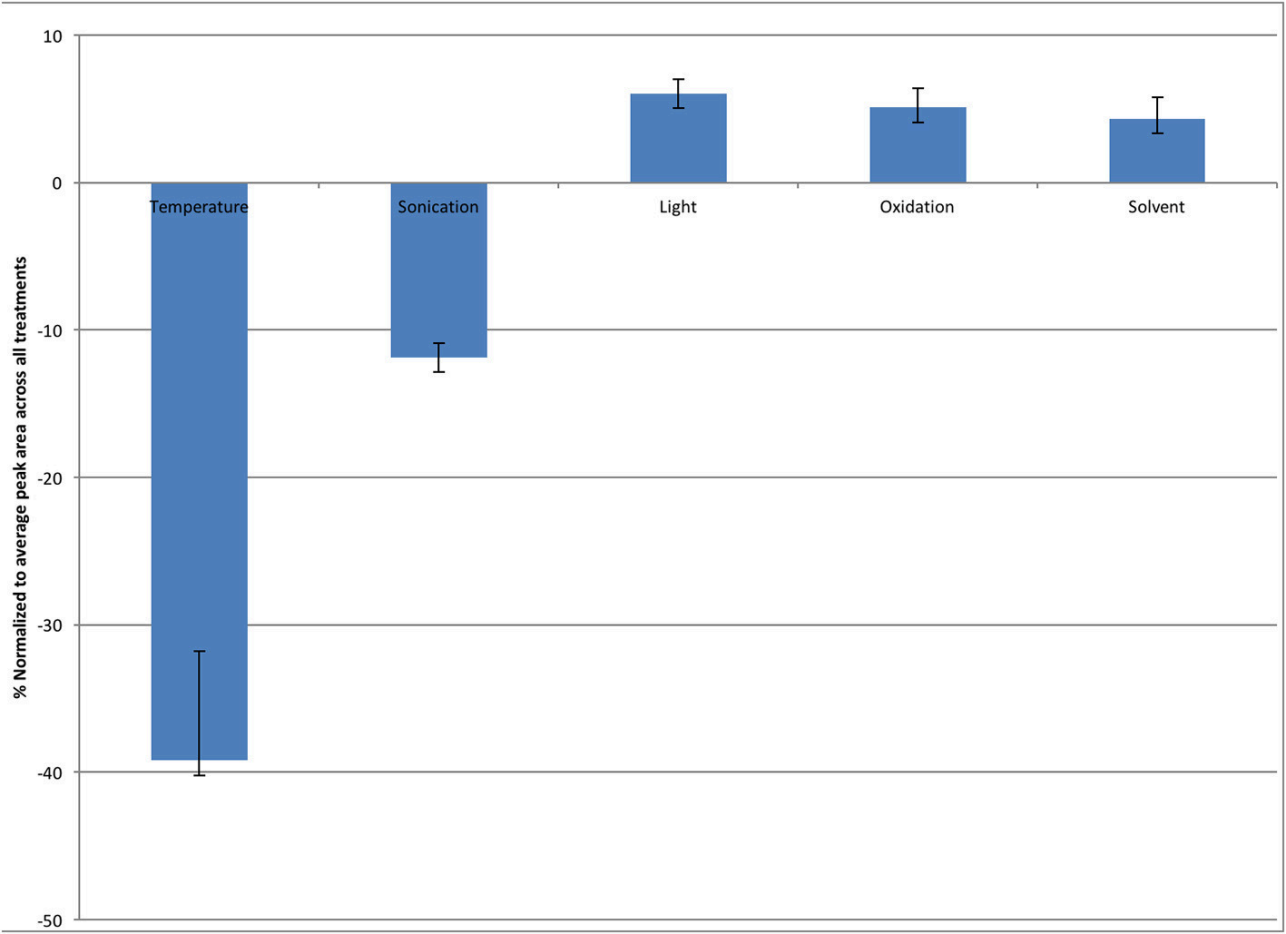
**Effect of varying extraction conditions on the stability of melatonin in solution, expressed as the difference between amount remaining at the high and low levels of factors**. Error bars represent standard error, *n* = 3.

Youden trials are factorial designs which are generally utilized to test the robustness of a method, and determine the level of variability which can exist in a particular method before the results are effected. As such two extreme values for likely conditions which a sample may be subjected to: a high and a low value are utilized and effects can then be measured (Karageorgou and Samanidou, 2014). In this case the Youden trial was run to investigate the stability of melatonin during the extraction protocol found that light, oxygen exposure (oxidation) and solvent concentration were not significant factors contributing to melatonin degradation. Temperature had a significant impact on melatonin concentration remaining in samples, while sonication had a nominal effect (Figure 3).

**Figure 4:**
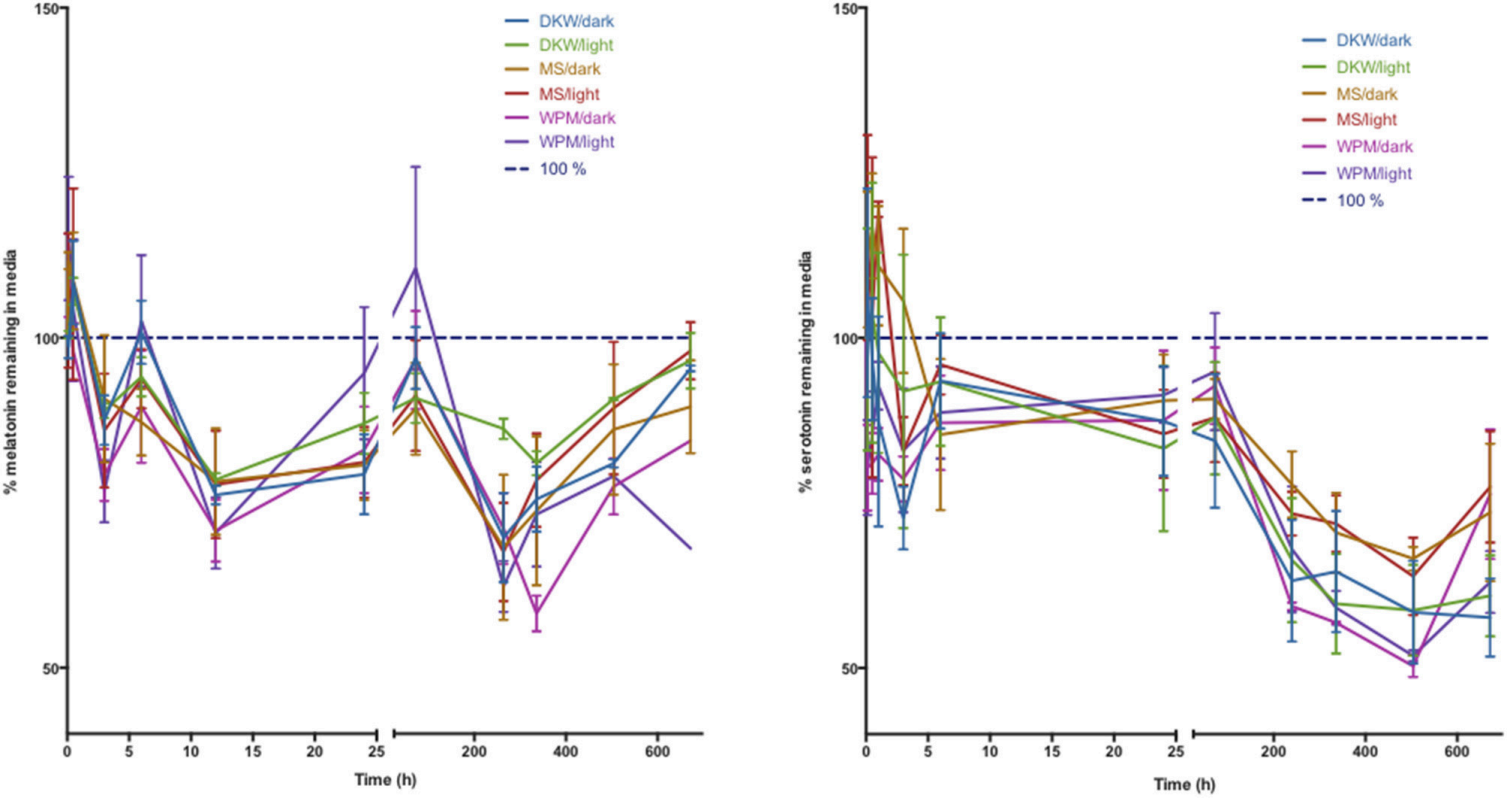
**Stability of melatonin (left) and serotonin (right) in three types of plant tissue culture medium**. DKW—Driver Kuniyuki walnut; MS—Murashige and Skoog; WPM—woody plant medium. Dark indicates 24 h darkness, light indicates a 16 h photoperiod. Initial media concentration of melatonin and serotonin was 25 μM.

Investigation of the stability of melatonin and serotonin found that compounds remained relatively stable across the first 10 days in media with values declining up to a 10% loss for melatonin at day 28 and losses of up to 40% at 28 d for serotonin. There was no significant difference in the trends observed for any of the three media types: WPM, DKW, and Murashige and Skoog and no difference between culture boxes stored in the light or in complete darkness of the 28 d period (Figure 4).

## Discussion

Melatonin is increasingly being recognized as an important regulator of plant growth, development and adaptation (Erland et al., 2015). As such there is a rapidly growing body of knowledge examining the roles melatonin plays in plants, many of which employ controlled environment systems and in particular *in vitro* culture systems, many of which are helping to solidify the role of melatonin in plant processes. As both the receptors and mechanisms underlying the functions of melatonin in plants while an active area of research are still poorly understood, *in vitro* systems offer a valuable platform for their investigation (Lee and Back, 2016; Sanchez-Barcelo et al., 2016; Shi et al., 2016; Wei et al., 2016). One such important strategy, which has helped to confirm for example the importance of calcium signaling in melatonin responses in several species, is the inclusion of inhibitors in plant medium (Murch et al., 2001; Jones et al., 2007; Ramakrishna et al., 2009, 2011). The *in vitro* culture system also offers a unique opportunity compared to greenhouse and field trials, in that treatment conditions can be closely monitored and allow for treatment of many plants, and all cultures are maintained in aseptic conditions. This of particular value with respect to the indoleamines, due to their ubiquitous production across kingdoms, therefore any microbial contamination could confound important results.

As the interest in melatonin continues to rise, many labs require not only practical and effective platforms via which to study the physiological effects of melatonin but also assays by which to determine the actual quantities of melatonin in a particular sample. Though melatonin has now been examined in many systems, including in culture, there still remains inconsistency in the literature as to the quantities of melatonin in individual plants and the methods by which to extract it. This is well illustrated in Table 1 which summarizes some of the many extraction protocols which have been utilized. Times to complete extraction range from <15 min to over 16 h, and in most cases specific times to complete the entire extraction process are not specifically mentioned, with the reader left to presume extraction times based on the time for individual steps such as shaking, sonication or drying. The relevance of this inconsistency in extraction conditions, is also reflected in several reports on the stability of melatonin. Though several reports have found melatonin to be stable in aqueous solution over long periods of time regardless of pH (e.g., 5–12) and storage temperatures (e.g., 4 vs. −70°C), still others have suggested that melatonin may undergo degradation under particular conditions such as light exposure (Cavallo and Hassan, 1995; Daya et al., 2001; Maharaj and Dukie, 2002). The majority of these studies have, as noted, been performed in aqueous solution and with a view toward mammalian systems. As plant systems have highly complex phytochemical environments, this level of complexity may suggest that further investigation is required specifically addressing the unique challenges presented by the plant system. Though the issue of the presence of melatonin isomers in plants has been raised by several reports (Tan et al., 2012), and represents an important field of study, the total melatonin content, or the sum of all melatonin isomers present in a sample, is the most commonly reported value across studies, and represents sufficient information for many basic plant physiological studies. Additionally, by considering only the total content, this allows for many more labs, beyond those with the specialized equipment and analytical background required for isomer determination, to investigate melatonin content in plants.

In view of these inconsistencies, this study presents two noteworthy achievements. First it presents a simple, accessible and easy to implement protocol for the analysis of not just melatonin or serotonin in plant tissues, but also their three precursors: tryptophan, tryptamine, and NAS, and secondly it presents an in-depth investigation of the levels of melatonin and serotonin which persist in plant tissue culture medium under variable lighting conditions and with varying media composition.

Despite previously published literature suggesting that light is a major factor leading to the degradation of melatonin, this study found that heat and to a lesser extent sonication had the greatest effect on the stability of melatonin, as sonication produces heat, even when sonication is performed in an ice bath, these two factors are interrelated emphasizing the impact of temperature on the stability of melatonin (Figure 3) (Maharaj and Dukie, 2002).

The method provided in this report is robust, reproducible and relatively simple, eliminating many factors which provide opportunities for loss of analyte, which is of particular importance, given concerns about the stability of melatonin in previous reports. Additionally, as interest in the mechanism behind melatonin's action in plants continues to grow, there is a need for easy to implement methods which provide reproducible results for not just melatonin, but its biosynthetic precursors as well. Accurate quantification of these compounds will allow for more in-depth studies into this important phytochemical signaling molecule.

This study has employed this method to address a significant question in melatonin investigations in plants. There are now many reports on the role melatonin plays in plant development, reproduction and survival of biotic and abiotic stresses (Erland et al., 2015; Reiter et al., 2015; Hardeland, 2016). Many investigations employ treatment of plants with melatonin in a liquid dose, or in systems such as plant tissue culture in liquid or solid medium. In particular, *in vitro* culture experiments involve the storage of plants under light conditions and relatively warm temperatures for extended periods of time with data collection often happening after days or weeks. The actual levels of melatonin or serotonin in this medium, however, have never been determined. Due to previous reports of the instability of melatonin under light and a negative effect of temperature as reported in this manuscript, a study was undertaken to investigate the actual exposure concentrations for melatonin in *in vitro* culture systems. This is particularly salient as significant or immediate decreases in indoleamine content could lead to much lower exposure concentrations than are reported and present an artificially inflated active concentration. Surprisingly, both serotonin and melatonin were found to be stable in three common media types tested for 10 days of culture. This time-frame is important as developmental patterns are often established early-on in *in vitro* cultured plants and tissues. Many experiments, however, use 21 and 28 days as convenient points for data collection as plant organs are generally sufficiently developed by this time to allow for accurate measurement. It was therefore important to determine if the concentrations in medium are sustained over the entire experiment. Though variability is present in media data, possibly due to box to box differential degradation across culture vessels, and variability introduced during sample collection, these fluctuations do not significantly change the observed trend. Serotonin showed up to a 40% loss in concentration after 28 days, surprisingly, melatonin only showed a 10% decrease in concentration.

Many plant growth experiments use different growth medium compositions varying sources of nitrogen, iron, and other important nutrients to support robust and normal plant growth. It was hypothesized that these different macro- and micro-nutrient compositions may have an effect on stability, if losses were due to a chemical interaction with media constituents (Murashige and Skoog, 1962; McCown and Lloyd, 1981; Driver and Kuniyuki, 1984). The results shown in Figure 2, however, show that there was not any difference in the degradation trend between three commonly used media types. Furthermore, it was theorized that light exposure would have a significant impact on media concentrations of melatonin and serotonin. Again, however, it was found that light levels (16 h photoperiod vs. complete dark) had no significant effect on concentrations, suggesting that photooxidation or photo-degradation is not a significant factor in the design of experiments. This is particularly important as many reports have utilized diverse culture vessels ranging from clear glass test tubes to black plastic pots and employ different lighting conditions ranging from ambient light in a greenhouse to strictly controlled light spectra (Erland et al., 2015). These results eliminate vessel color, wavelength or light conditions as an important variable in these experiments and provide further confidence moving forwards.

The data presented in this study are relevant as they address an important but previously un-investigated variable in the design of plant physiology experiments in plant culture systems. Additionally, they validate many previous studies which have been published investigating the important roles of melatonin across diverse plant species and in response to changing conditions (Erland et al., 2015; Reiter et al., 2015; Hardeland, 2016).

In summary, a validated method which allows for accurate, sensitive, and reproducible quantification of melatonin, and its biosynthetic precursors: serotonin, tryptophan, NAS, and tryptamine, was determined. This method was found to be robust in the analysis of these compounds across diverse plant species and tissue types. Additionally, measurement of melatonin and serotonin via this method in plant tissue culture medium found that neither light nor media composition had an effect on stability of melatonin or serotonin in these systems. Both melatonin and serotonin were found to be stable in medium across 10 days and losses after 28 days only reached 10 and 40% of the initial concentration respectively. These results pave the way for future in depth experiments examining the roles of melatonin and its precursors in both basic scientific investigations of plant physiological processes, and industrial applications such as micropropagation and cryo-banking.

## Author contributions

All authors (LE, AC, AJ, and PS) participated in experimental design, manuscript preparation and agree to be accountable for all aspects of the work and provided final approval of the version to be published; LE conducted experiments and performed data analysis; AC assisted in conducting experiments; PS was responsible for study conception; LE, PS, and AJ participated in interpretation of data.

## Funding

This research was supported by grants from the Gosling Foundation through the Gosling Institute for Plant Preservation and the National Sciences and Engineering Research Council (NSERC) [grant number 46741] of Canada.

### Conflict of interest statement

The authors declare that the research was conducted in the absence of any commercial or financial relationships that could be construed as a potential conflict of interest.

## Abbreviations

DKW: Driver and Kuniyuki walnut
ECD: electrochemical detector
ELISA: enzyme linked immunosorbent assay
ESI: electrospray ionization
FLD: fluorescence detection
LLOQ: lower limit of quantification
LN: liquid nitrogen
LOD: limit of detection
M: mechanical grinding
m/z: mass to charge ratio
MS: mass spectrometry
NAS: N-acetylserotonin
RSD: relative standard deviation
RT: room temp
SIR: single ion recording
SJW: St. John's wort
SPE: solid phase extraction
TLC: thin layer chromatography
ULOQ: upper limit of quantification
UPLC-MS: ultra performance liquid chromatography
UV: ultra violet detection
WPM: Lloyd and McCown's woody plant medium.
